# A Novel Genetic System Based on Zinc Finger Nucleases for the Identification of Interactions between Proteins *In Vivo*

**DOI:** 10.1371/journal.pone.0085650

**Published:** 2013-12-31

**Authors:** Ling Wang, Kun Xu, Juan Lin, Simin Shao, Tingting Zhang, Huarong Xu, Zehui Wei, Zhiying Zhang

**Affiliations:** College of Animal Science & Technology, Shaan'xi Key Laboratory of Molecular Biology for Agriculture, Northwest A&F University, YangLing, Shaan'xi, P. R. China; Huazhong University of Science and Technology, China

## Abstract

Yeast two-hybrid (Y2H) methods are powerful tools for detecting protein–protein interactions. The traditional Y2H method has been widely applied to screen novel protein interactions since it was established two decades ago. The high false-positive rate of the traditional method drove the development of modified Y2H systems. Here, we describe a novel Y2H system using zinc-finger nucleases (ZFNs). ZFNs contain two functional domains, a zinc-finger DNA-binding domain (ZFP) and a non-specific nuclease domain (FokI). In this system, the bait is expressed as a fusion protein with a specific ZFP, and the prey is fused to the FokI. A reporter vector is designed such that the ZFN target site disrupts the *Gal4* open reading frame. By transforming the three plasmids into a yeast strain (AH109), the interaction between the bait and prey proteins reconstitutes ZFN function and generates the double-strand break (DSB) on its target site. The DNA DSB repair restores Gal4 function, which activates the expression of the four reporter genes. We used p53-SV40LT interacting proteins to prove the concept. In addition, 80% positive rate was observed in a cDNA screening test against WDSV orfA protein. Our results strongly suggested that this Y2H system could increase screening reliability and reproducibility, and provide a novel approach for interactomics research.

## Introduction

Protein–protein interactions are involved in all biological processes. The exploration of these interactions aims to characterize intricate protein networks and functions in living cells. Yeast two-hybrid (Y2H) methods represent robustly genetic and biochemical approaches to profile the protein interactome [1]. Various Y2H methods have been developed to investigate protein interactions, and they have shown success in screening novel protein interactions [2-5]. The classical Y2H system is based on expressing a pair of proteins fused to a Gal4 DNA-binding domain (DBD) and Gal4 transcriptional activation domain (AD). In the presence of fusion protein interactions, Gal4 is reconstituted, which drives reporter gene expression [6]. This classical Y2H system was widely used in cDNA library screens. However, because of non-specific interactions, the generation of false positives is one major limitation of this system [7,8]. Therefore, various Y2H systems are expected to screen for novel protein–protein interactions, to explore global or targeted interactomics.

Zinc finger nucleases (ZFNs) are powerful artificial enzymes that are widely used to target specific DNA sequences in various species [9-13]. The specific ZFNs could generate double strand breaks (DSBs) at target sites, which would be repaired by homologous recombination (HR) or non-homologous end joining (NHEJ) *in vivo* [14] . In previous reports, ZFNs were customized to target genes of interest [15]. The two components of ZFNs, a zinc finger DNA-binding domain (ZFP) and a non-specific nuclease domain (FokI), were tandem and expressed as fusion proteins. FokI forms a dimer to cleave DNA.

Here, we describe a novel Y2H system based on ZFNs to identify protein–protein interactions. This new system uses ZFP and FokI as bait and prey, respectively, and a non-functional (ZFN target disrupted) *Gal4* is used as an intermediate reporter gene. The three plasmids are expressed in the AH109 yeast strain, which contains four integrated reporter genes that are driven by the Gal4 transcription factor. The interaction of bait and prey reconstitutes ZFN function, leading to a DSB at the ZFN target site located in the middle of the *Gal4* gene. The DSB promotes the DNA repair process, which in turn restores the function of the Gal4 transcription factor. Consequently, the functional Gal4 activates the four integrated reporter genes. This novel approach has a higher sensitivity as it amplifies the protein interaction signal via reconstitution of transcription factor Gal4. In addition, unlike traditional Y2H method, the bait protein can be any cellular protein, including proteins containing transcriptional domain. 

## Materials and Methods

### Construction of plasmids

Plasmids used in this study are listed in Table 1. The construction and amplification of plasmids were performed in *Escherichia coli* DH5α according to the manufacturer’s instructions. All plasmids were confirmed by sequencing. Constructions of novel plasmids to this study are summarized below. More information and maps of these plasmids are provided in the supplemental material.

**Table 1:** Plasmids used in this study.

Gene	Plasmid	Description	Source
FokI	pST1374		Addgene
BCR-ZFP	pGP-FB		Addgene
WDSV orfA	pcDNA-orfA		
Human E2F5	pcDNA-E2F5		
SV40LT	pGADT7-T	*LEU2* 2μm	Clontech
P53	pGBKT7-53	*TRP1* 2μm	Clontech
Gal4	pADH-Gal4	*KanMX4* CEN	This study
Gal4-MCS	pADH-Gal4-MCS	*KanMX4* CEN	This study
BCR binding sites	pADH-Gal4-BS	*KanMX4* CEN	This study
BCR-ZFN	pBCRZFN	*LEU2* 2μm	This study
ZFP-P53-FokI	pBCRZFN-53	*LEU2* 2μm	This study
ZFP-SV40 LT-FokI	pBCRZFN-LT	*LEU2* 2μm	This study
	pRSADH	*TRP1* 2μm	This study
BCR-ZFP	pRS-ZFP	*TRP1* 2μm	This study
ZFP-SV40LT	pRS-ZFP-LT	*TRP1* 2μm	This study
ZFP-WDSV orfA	pRS-ZFP-A	*TRP1* 2μm	This study
	pYEADH	*LEU2* 2μm	This study
FokI	pYE-FokI	*LEU2* 2μm	This study
FokI-p53	pYE-Fok-53	*LEU2* 2μm	This study
FokI-E2F5	pYE-Fok-E2	*LEU2* 2μm	This study

pBCRZFN-53: A truncated murine p53 gene (amino acids 72–390) was amplified from pGBKT7-53 (Clontech, Cat. NO. 630489) by PCR with primers p53-BHF(5'-CGCGGATCCCCTGTCACCGAGACCCCTG-3') and p53-SpeR (5'-GGACTAGTCTGTCTGAGTCAGGCCCCACT-3') and cloned into *Bam*HI/*Spe*I sites of the pBCRZFN vector, resulting in the pBCRZFN-P53 plasmid (Figure S1).

pBCRZFN-LT: A truncated SV40 large T-antigen (SV40LT) (amino acids 87–708) was amplified from pGADT7-T (Clontech, Cat. NO. 630489) by PCR with primers LT-BHF(5'-CGCGGATCCGGAACTGATGAATGGGAGC-3') and LT-SpeR(5'-GGACTAGTACTGTTTCAGGTTCAGGGGGAG-3'), and cloned into *Bam*HI/*Spe*I sites of the pBCRZFN vector, resulting in pBCRZFN-LT (Figure S1).

pRS-ZFP: Three-zinc finger protein specifically targeting a 9-bp BCR-ABL sequence was amplified by PCR from plasmid pGP-FB (Addgene, Cambridge MA, the U.S.) using a forward primer ZFF(5'-CCCACTAGTACAATCAACTCC ATGGGACC-3') with a *Spe*I site and a reverse primer (5'-CCCGAATTCCCT CAGGTGGGTTTTTAGG-3') with an *Eco*RI site and cloned into pRS-ADH to generate pRS-ZFP (Figure S2).

pRS-ZFP-LT: SV40LT was obtained by *Eco*RI/*Xho*I double digestion of pGADT7-T and was subcloned into pRS-ZFP, resulting in the plasmid pRS-ZFP-LT (Figure S2).

pRS-ZFP-A: The walleye dermal sarcoma virus (WDSV) orfA gene was obtained by *Eco*RI/*Xho*I double digestion of pcDNA-orfA and was subcloned into the *Eco*RI/*Xho*I sites of the pRS-ZFP vector, resulting in the pRS-ZFPA plasmid (Figure S2).

pYE-FokI: The FokI non-specific catalytic domain was amplified by PCR from pST1374 (Addgene) using the primers Fok-F (5'-CCTCTAGAATGGGACCTAAGAAAAAGAGGAAGGTGCAACTAGTCAAAAGTGAACTGGAGG-3'), introducing an *Xba*I site, and Fok-R (5'-CCGGATCCAAAGTTTATCTCGCCGTTA-3'), introducing a *Bam*HI site, and cloned into the *Xba*I/*Bam*HI sites of the pYEADH vector, resulting in the plasmid pYE-FokI (Figure S3).

pYE-Fok-53: A truncated p53 gene was generated by *Eco*RI/*Sal*I double digestion of pGBKT7-53 and subcloned into pYE-FokI to generate the pYE-Fok-53 plasmid, which encodes FokI and p53 fusion protein (Figure S3).

pYE-Fok-E2: The human E2F5 gene was obtained by *Sac*II/*Nco*I double digestion of the pcDNA-E2F5 vector and was subcloned into the *Sac*II/*Nco*I sites of pYE-FokI, resulting in the pYE-Fok-E2 plasmid (Figure S3).

pADH-Gal4-BS: Two complementary oligos bearing BCR-ABL binding sites, BS-F (5'-GGCCGCGGCTTCTGCTGATAAGCAGAAGCCG-3') and BS-R (5'-GATCCGGCTTCTG CTTATCAGCAGAAGCCGC-3') were annealed and cloned into the *Not*I/*Bam*HI sites of the pADH-Gal4-MCS vector, resulting in the pADH-Gal4-BS plasmid, in which the *Gal4* gene was interrupted by the binding sites (Figure S4).

### Yeast strain, medium, and manipulations

The host *Saccharomyces cerevisiae* strain AH109 used in this study has the genotype of *MATa, trp1-901, leu2-3, 112, ura3-52, his3-200, Gal4Δ, gal80Δ, LYS2::GAL1*_*UAS*_ *-GAL1*_*TATA*_ *-HIS3, GAL2*_*UAS*_ *-GAL2*_*TATA*_ *-ADE2, URA3::MEL1*_*UAS*_ *-MEL1*_*TATA*_ *-LacZ MEL1* (Clontech, Cat. NO.630489). *S. cerevisiae* AH109 harbors four integrated reporter genes under the control of distinct Gal4-responsive upstream activating sequences (UASs) and TATA boxes. Another yeast strain, Y187, is the genotype of *Matα, ura3-52, his3-200, trp1-901, leu2-3, 112, Gal4Δ, gal80Δ, met-URA3::GAL1*_*UAS*_ *-GAL1*_*TATA*_ *-LacZ MEL1* (Clontech). The yeast rich medium YPD contains 1% yeast extract, 2% peptone, 2% glucose, and 1.5% bacto agar when prepared for plates. Synthetic medium SD contains 0.67% yeast nitrogen base, 0.06% complete dropout amino acid mixture (Clontech, Cat. NO.630428), and 2% glucose. Auxotrophic selection plates are SD supplemented with 1.5% bacto agar, 50 μg/mL G418 and appropriate dropout amino acid mixture. Yeast media preparation and growth were performed by standard techniques [16].

### Yeast transformation and spotting

Quick and easy yeast transformation was performed by the lithium acetate method as previously described [17]. Transformants were grown at 30°C for at least 3 days on appropriate SD plates. The transformation efficiency was detectable on non-selective plates with histidine and adenine. Transformants survived on selective plates without histidine and adenine are positive candidate colonies. Several colonies were transferred to the appropriate liquid SD medium for further analysis. For spotting, when the cell culture optical density was about OD_600_ = 1.0, five microliters of different dilutions (1:1, 1:10, 1:100, and 1:1000) were spotted onto non-selective and selective media and maintained at 30°C for at least three days.

### Reporter yeast strain construction

The unique reporter vector pADH-Gal4-BS has an autonomously replicating sequence (ARS), a yeast centromere (*CEN*), and a selectable marker (*KanMX4*). First, the reporter plasmid pADH-Gal4-BS was transformed into AH109, and successful transformants were grown on YPD plates with 50 μg/mL G418 at 30°C for three days. Then, two identical colonies carrying low copy reporter plasmids were grown in liquid YPD medium with 50 μg/mL G418 for the preparation of co-transformation with bait and prey expression vectors. Ultimately, reporter strains harboring both bait and prey plasmids were grown and selected on either non-selective or selective medium.

### Relative β-galactosidase activity assay

One of the reporter genes, *LacZ*, encodes β-galactosidase, which is not secreted by the yeast cells. β-galactosidase activity was analyzed by the ortho-nitrophenyl- β-galactosidase (ONPG) assay as described previously [18]. To measure the relative β-galactosidase activity, *LacZ* expression driven by the wildtype *Gal4* in the plasmid pADH-Gal4 (Figure S4) was set arbitrarily to 100% for the assay.

### Fusion proteins expression validation

To determine ZFP- and FokI- fusion proteins expression, whole proteins were extracted from yeast culture with glass beads. And ZFP-fusion proteins ZFP-LT and ZFP-orfA with a flag tag were detected by western blot with flag tag monoclonal antibodies (abcam, ab18230). And FokI-E2F5, FokI-p53 fusion proteins without tags were detected by western blot using specific anti-human E2F5 antibody (abcam, ab59769) and anti-p53 antibody (abcam, ab90363), respectively. 

### cDNA library screening

For the construction of the the human cDNA library expression plasmid, the cDNA was excised from the human library plasmid pAP3neo-cDNA (TaKaRa, Code NO. 9505) by *Xba*I/*Not*I and subcloned into pYE-Fok-MCS, resulting in pYE-Fok-cDNA. The library represents about 1×10^6^ independent colonies. The cDNA library pYE-Fok-cDNA as prey vector was transformed into yeast strain Y187 using the lithium acetate method [25]. In brief, 10 μg library plasmid and 0.5 μg plasmid pYE-Fok-E2 mixture were transformed into Y187 with large-scale transformation. Transformants were plated onto SD-Leu medium and incubated at 30°C for three days. Colonies on SD plates were harvested and grown in liquid SD-Leu medium at 30°Cfor three days. 

Before mating, reporter strain AH109 containing both reporter vector pADH-Gal4-BS and bait vector pRS-ZFP-A and yeast Y187 harboring library vector were grown in YPD rich medium overnight. Then cultures of AH109 and Y187 were harvested and transferred into a 2L flask with 10ml 2×YPD medium, and incubated at 30°C for 24 hours, slow shaking with 40rpm. Then the mating culture was plated on non-selective plates with 1/10, 1/100 dilutions to test mating efficiency. The remainder of culture was plated on selective medium with 200 μL per 150mm plate and incubated at 30°C for 3-8 days.

Colonies survived on selective media plates were transferred into liquid SD medium. Plasmids were recovered from positive colonies, and library plasmids were isolated via colony PCR using primers LeuF and LeuR. *Bam*HI/*Xho*I double digestion of recovered positive library plasmids was used for analysis insert size, and sequencing insert fragments for further confirmation. 

## Results

### Design and construction of the ZFN-Y2H system

Our novel approach to the identification of protein–protein interactions takes advantage of ZFN technology. ZFNs are composed of the DNA-binding domain of ZFP and the non-specific catalytic domain of FokI; two domains that enable ZFNs to target specific DNA sequences *in vivo* and *in vitro*. In addition, ZFNs are generally designed to target genes of interest in various species and human cells, in which ZFPs and FokI are expressed as a fusion protein.

**Figure 1:**
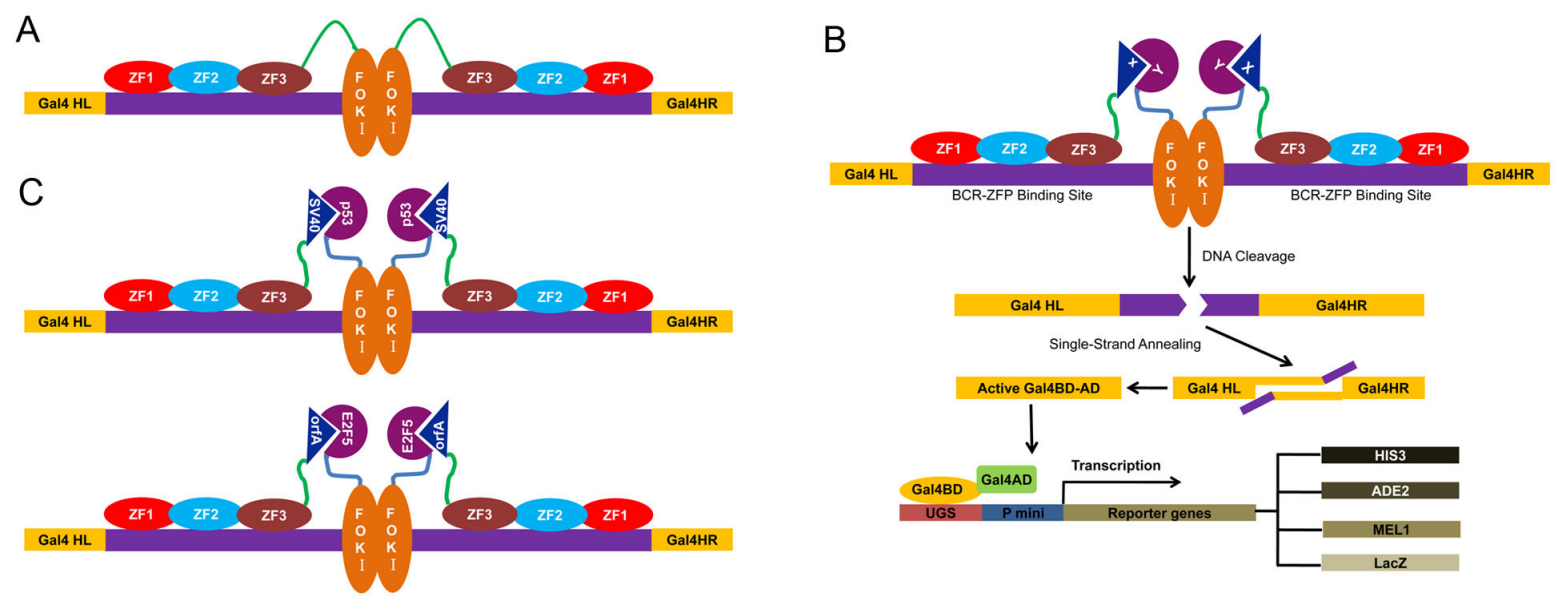
Schematic diagram describing the zinc finger nuclease yeast two-hybrid (ZFN-Y2H) system to identify protein–protein interactions. (A) Traditional ZFNs are composed of a fused protein of a zinc finger DNA-binding domain (ZFP) and a non-specific nuclease domain (FokI), in which ZFPs recognize binding sites and recruit dimers of FokI to cleave DNA specifically and highly efficiently. (B) A known protein partner X is expressed as a fusion protein with BCR-ZFP for the bait, and another known protein Y or cDNA library is fused with FokI as the prey. When proteins X and Y interact with each other, FokI is recruited as a dimer to cleave a target site within Gal4 and generate double-strand breaks (DSBs), which could be repaired by single-strand annealing (SSA). Thus, a functional Gal4 DNA-binding domain (DBD) and activation domain (AD) are restored, which drives the expression of reporter genes. (C) Illustration of p53-SV40LT and WDSV orfA-E2F5 interactions in this Y2H system.

Based on the simple idea that ZFP and FokI are two separable domains that can be either linked together by a short linker peptide (Figure 1A) or brought together through protein–protein interactions (Figure 1B), they can serve the purpose of bait and prey. After they are brought together, instead of activating gene transcription as Gal4 would do, reconstituted ZFNs will recognize and cleave a specific DNA sequence. We engineered a system where this specific DNA was placed within the *Gal4* gene on another reporter vector. The reporter vector contains a 24-bp BCR binding site (BS) flanking the repeat sequences in the Gal4-DBD domain, in which stop codons would abolish Gal4’s ability to activate transcription. Therefore, only successfully reconstituted ZFNs could cut binding sites to generate double strand breaks (DSB). Subsequently, DSBs trigger cellular single strand annealing (SSA) repair pathway to restore wild type Gal4 to activate the transcription of reporter genes.

Here, we took advantage of a ZFP target BCR-ABL site, in which three zinc fingers could bind the DNA sequence 5'-GCAGAAGCC-3'[19]. Thus, the corresponding target DNA sequence in the reporter vector contained two inverted BCR-ABL binding sites flanked by two *Gal4* repeat sequences. Two well-recognized protein pairs, murine p53-SV40LT [20], and WDSV orfA–human E2F5 [21] were taken as protein X and Y to confirm the hypothesis that the interaction between proteins X and Y could reconstitute functional ZFNs (Figure 1C).

### Proof-of-concept

**Figure 2:**
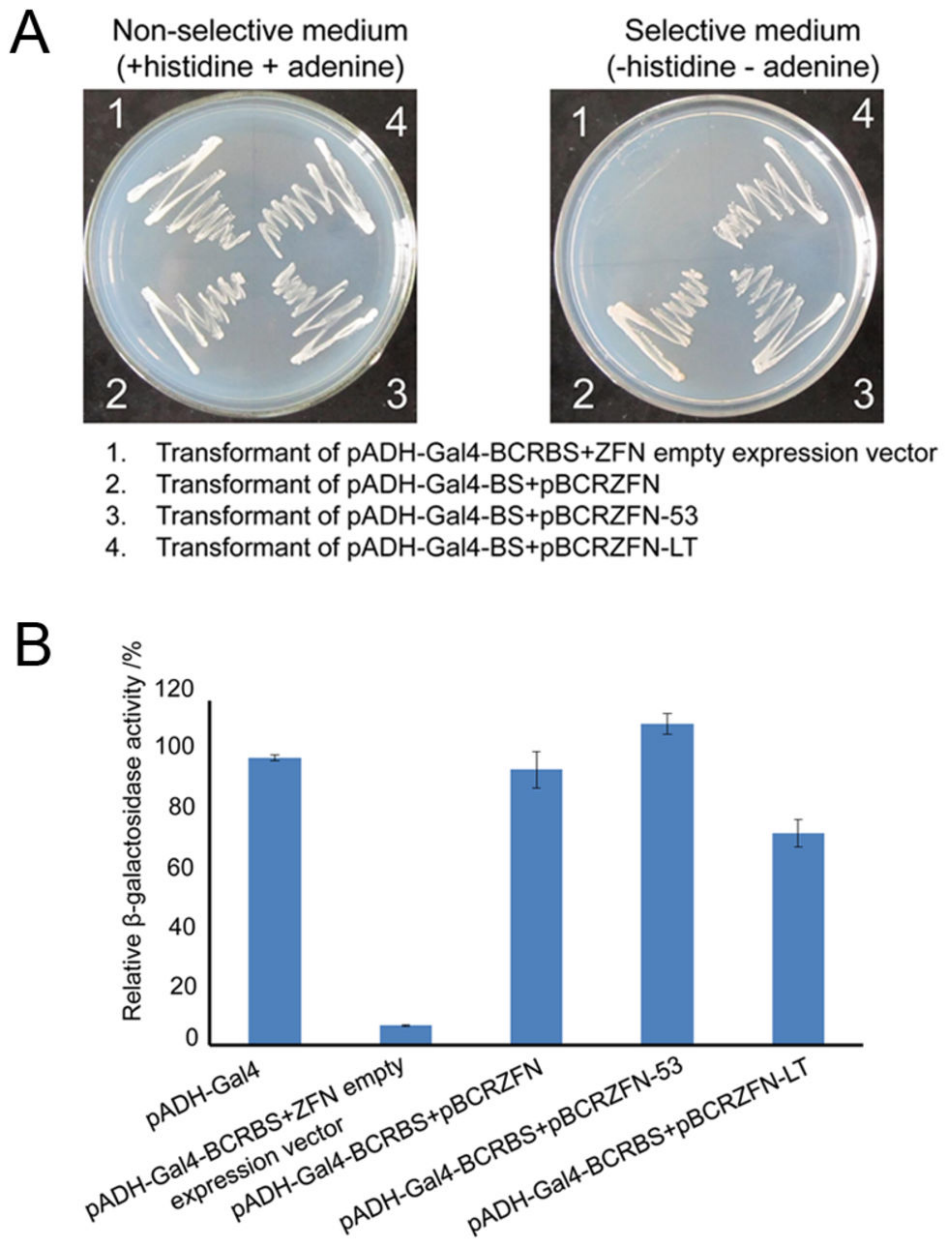
Detection of the function of ZFNs with different distances between the ZFP and FokI domains. (A) Detection of auxotrophic selection pressure. Yeast strain AH109 was transformed with a reporter plasmid and different ZFNs. Transformed yeast colonies were spotted? on non-selective medium and selective medium, and they were grown at 30°C for 3 days. (B) Relative β-galactosidase activity assay. Three independent transformants were assayed in each group. The transformants of pADH-Gal4 were the positive control, and β-galactosidase activity was set to 100%. Error bars represent the standard deviation from 3 independent experiments.

First, the major difference between our engineered ZFNs and traditional ZFNs is that FokI and ZFP are separated and connected by two relatively large protein domains in our system instead of a short linker. Thus, whether the modified ZFNs would work well with the long distance between ZFP and FokI was a big concern. To test our system, ZFN expression vectors were transformed into a reporter strain carrying the plasmid pADH-Gal4-BS and selected on SD plates without histidine and adenine (Figure 2A). pBCRZFN-53 and pBCRZFN-LT express ZFP-P53-FokI and ZFP-LT-FokI fusion proteins, respectively. As a positive control, plasmid pBCRZFN encodes a traditional ZFN with a short linker between the ZFP and FokI domains, and transformation of a ZFN empty expression plasmid is taken as a negative control. Transformants of the ZFN empty expression vector could not survive on selective medium. In contrast, yeast cells expressing traditional and modified ZFNs exhibit efficient growth on selective media. Several colonies of each sample were grown in an appropriate liquid SD medium, and the relative β-galactosidase activity was measured (Figure 2B). Transformants maintaining an empty expression vector had a relative β-galactosidase activity of 6.84%, which was significantly lower than that of colonies containing pADH-Gal4. In contrast, the relative β-galactosidase activity of colonies expressing BCRZFN was 95.82%. The *LacZ* expression of transformants with modified ZFN expression vectors was relatively high, and their relative β-galactosidase activities were 111.80% and 73.81%. The data indicated that functional Gal4 was restored by the ZFN-induced DSB in the reporter plasmids. Importantly, these results strongly suggested that ZFNs could tolerate long distances between the ZFP and FokI domains.

A fundamental concept in this approach is that ZFP and FokI could not form a functional ZFN in the absence of protein interactions *in vivo*. To confirm this, we checked the growth behaviors of reporter cells expressing BCRZFP together with FokI. As expected, a large number of colonies grew on the non-selective SD plates, suggesting that transformation efficiency was high, but no colonies grew on plates lacking histidine and adenine. This illustrated that separated BCRZFP and FokI do not have the ability to cleave DNA at a target site like a functional ZFN (Figure 2).

**Figure 3:**
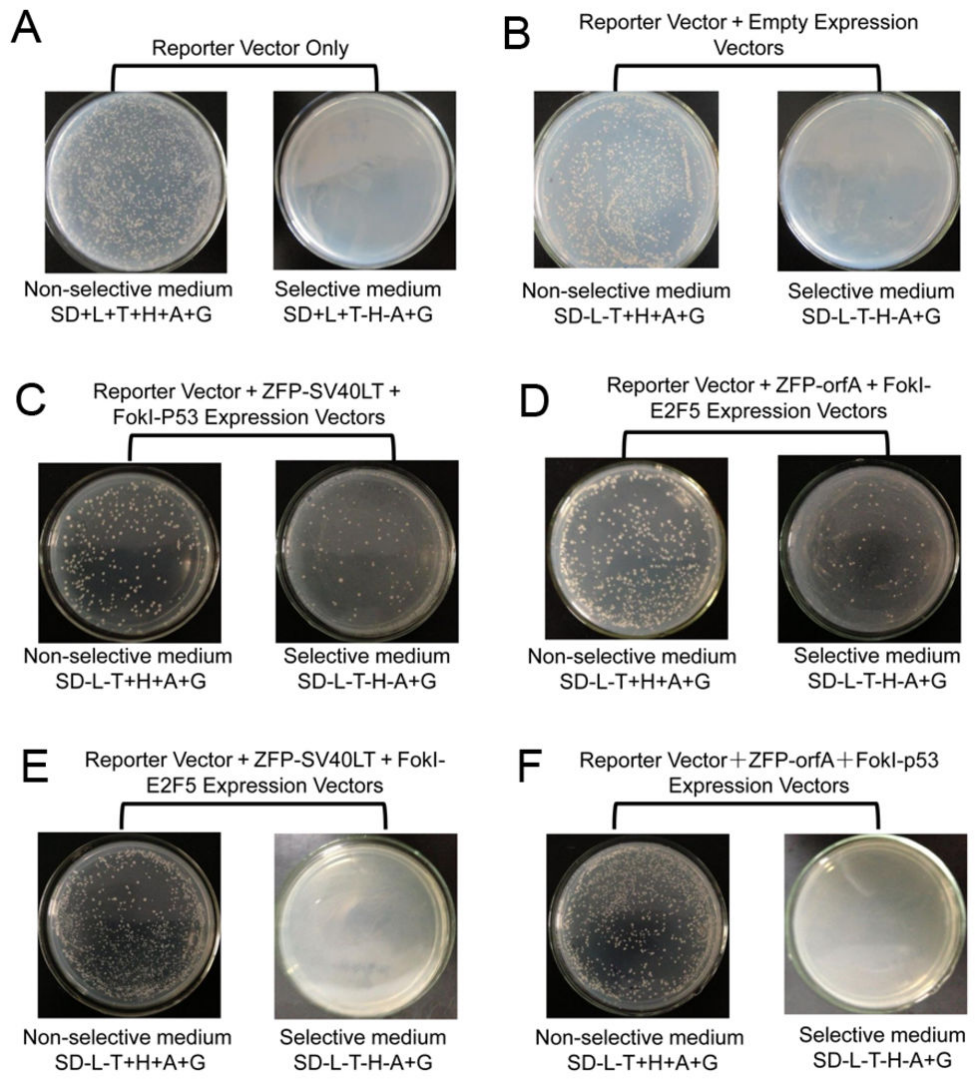
Confirmation of the ZFN-mediated protein identification system. Upon the interaction of well-known proteins fused with BCRZFP and FokI, ZFNs were functional and successfully cleaved DNA at target sites. (A) Transformants with the reporter vector only cannot survive on plates lacking histidine and adenine. In contrast, colonies were observed on plates with histidine, adenine, and G418. (B) Co-transformants pRS-ZFP and pYE-FokI empty expression vectors cannot survive on plates lacking histidine and adenine. (C) Transformants of the ZFP-SV40LT fusion protein and FokI-p53 fusion protein. Upon the interaction of p53 and SV40LT, ZFNs were reconstituted and bound the target binding site. Subsequently, colonies grew on plates lacking histidine and adenine upon the restoration of functional Gal4. (D) Co-transformation of BCRZFP-orfA and FokI-E2F5 expression vectors into reporter yeast cells. Transformants survived on plates lacking histidine and adenine. (E) Co-transformation of ZFP-SV40LT and FokI-E2F5 expression vectors into reporter yeast cells. (F) Co-transformation of ZFP-orfA and p53 expression vectors into reporter yeast cells. No colonies survived on selective plates when yeast co-expression of non-cognate proteins. No cross reactivity was observed between either SV40LT/E2F5 or orfA/p53. L, leucine; T, tryptophan; H, histidine; A, adenine; and G, G418.

To test the feasibility of this approach, we first tested the interaction between known interacting protein pairs. One pair is p53 and SV40LT , and another pair is WDSV orfA and E2F5, whose interaction was first found in our laboratory (data not shown). We constructed plasmids pRS-ZFP-LT, pYE-Fok-53, pRS-ZFP-A, and pRS-Fok-E2, and paired them with the report vector pADH-Gal4-BS. The co-transformation of these two expression plasmids into a reporter strain was performed as previously described. Transformants were grown on non-selective and selective media at 30°C for at least three days. Compared to the number of colonies on plates to detect the transformation efficiency, about 16.7% of transformants expressing BCRZFP-SV40LT and FokI-p53 survived without histidine and adenine (Figure 3C). Meanwhile, approximately 22.5% of transformants expressing BCRZFP-orfA and FokI-E2F5 fusion proteins grew on plates lacking histidine and adenine (Figure 3D). In addition, no colonies survived on selective plates co-transformed with either ZFP-orfA and FokI-p53 expression vectors or ZFP-SV40LT and FokI-E2F5 expression vectors (Figures 3E, 3F). These results demonstrated that specific protein interactions between p53 and SV40LT or orfA and E2F5 reconstituted FokI and ZFP as an efficient ZFN to target binding sites, and functional Gal4 was restored by SSA repair to drive the expression of His3 and Ade2 reporter genes. Also, no cross reactivity was observed when no-cognate proteins were expressed in reporter strains.

### Further confirmation of the ZFN-Y2H system with high stringency

**Figure 4:**
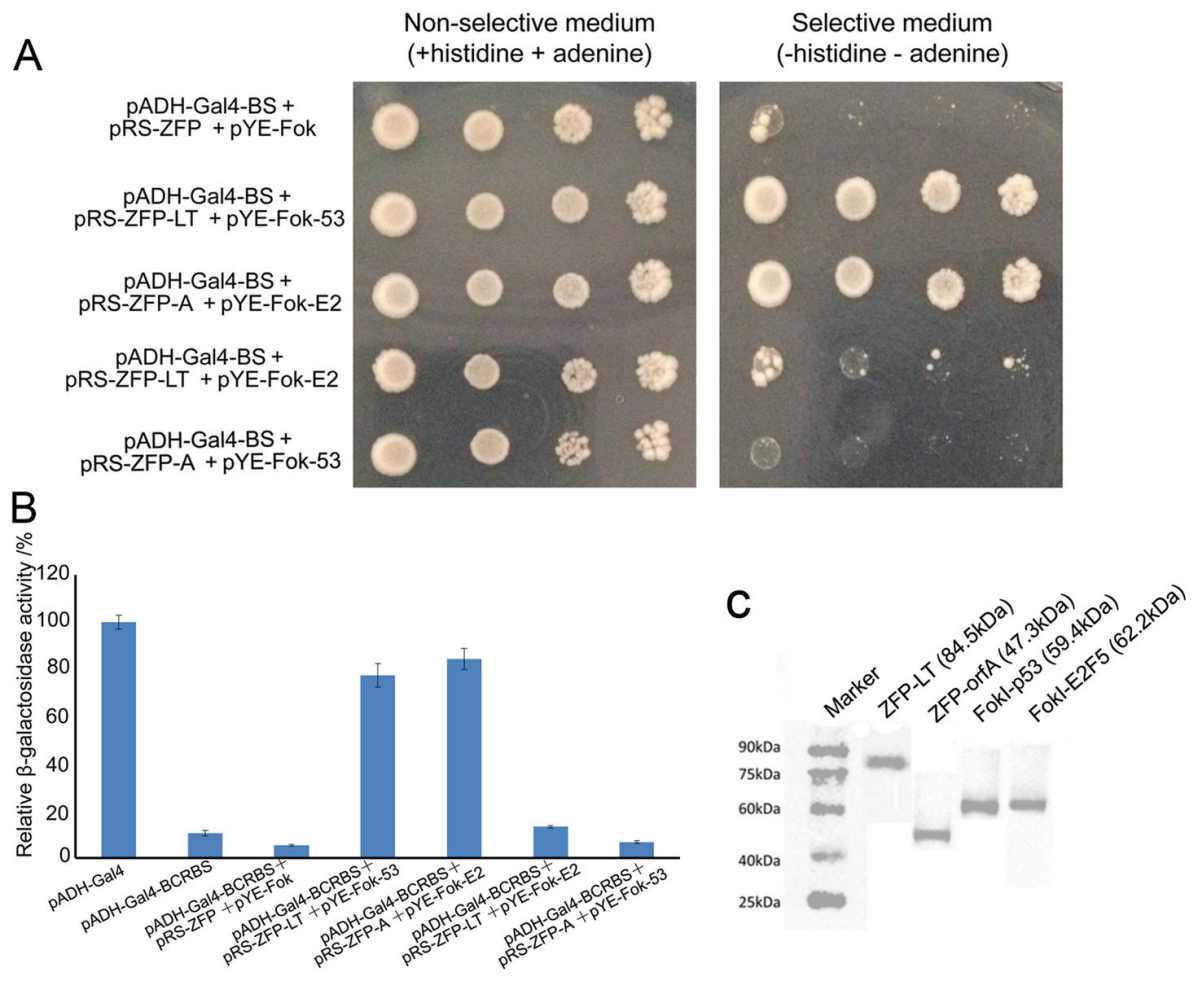
Further investigations of protein interactions. (A) Serial dilutions of yeast transformant colonies that were spotted on plates with histidine and adenine all showed robust growth. Under the auxotrophic selection pressure, colonies containing reporter vectors and empty expression vectors did not grow. In contrast, colonies containing reporter vectors and ZFP-p53 + FokI-SV40LT or ZFP-orfA + FokI-E2F5 protein pair expression vectors grew well. (B) Relative β-galactosidase activity assay. Three independent transformants were assayed in each group. Error bars represent the standard deviation from 3 independent experiments. (C) Western blot was performed to validate ZFP- and FokI- fusion proteins expression in yeast.

We took one step further to study the stability and robustness of our ZFN-mediated Y2H system. Several colonies from plates lacking histidine and adenine shown in Figure 3 were transferred into liquid SD medium lacking tryptophan and leucine and grown overnight. Then 5 μL of different dilutions was spotted onto appropriate plates (Figure 4). Colonies containing empty expression vectors could not survive on the selective medium plates, while colonies expressing either BCRZFP-LT/FokI-p53 or BCRZFP-orfA/FokI-E2F5 protein pairs were detectable on selective plates. These results confirmed the stability of colonies resulting from Figure 3C and 3D. Our system also passed the test of another reporter gene, *LacZ*. Several colonies were transferred into liquid medium for the relative β-galactosidase activity assay. Yeast cells containing either reporter vectors only or a reporter and two empty expression vectors had quite low background *LacZ* expression. In contrast, transformants of corresponding bait and prey expression vectors had a relatively high level of *LacZ* expression, and they had relative β-galactosidase activities of 77.36% and 84.35% (Figure 4B). However, the relative β-galactosidase activities in colonies that expressed ZFP-orfA/FokI-p53 and ZFP-SV40LT/FokI-E2F5 were 13.17% and 6.81%, respectively. Additionally, expression of these fusion proteins was detected by western blot via specific antibodies (Figure 4C). These data are in positively correlated with the results of nutrient pressure selection. Collectively, these results illustrated that the expression of reporter genes in yeast transformants was strongly dependent on the specific interactions of proteins X and Y, and no cross reactivity was indicated in this system.

### cDNA library test

To test the feasibility of this system for library screening, we tested the orfA protein to screen for E2F5 in a library. The cDNA expression library was constructed as FokI fusion, designated as pYE-Fok-cDNA. This cDNA library plasmid and FokI-E2F5 expression plasmid pYE-Fok-E2 mixture were transformed into Y187 as prey, and the reporter vector and ZFP-orfA expression plasmid were co-transformed into AH109 as bait. Yeast mating was performed as previously described. 

**Figure 5:**
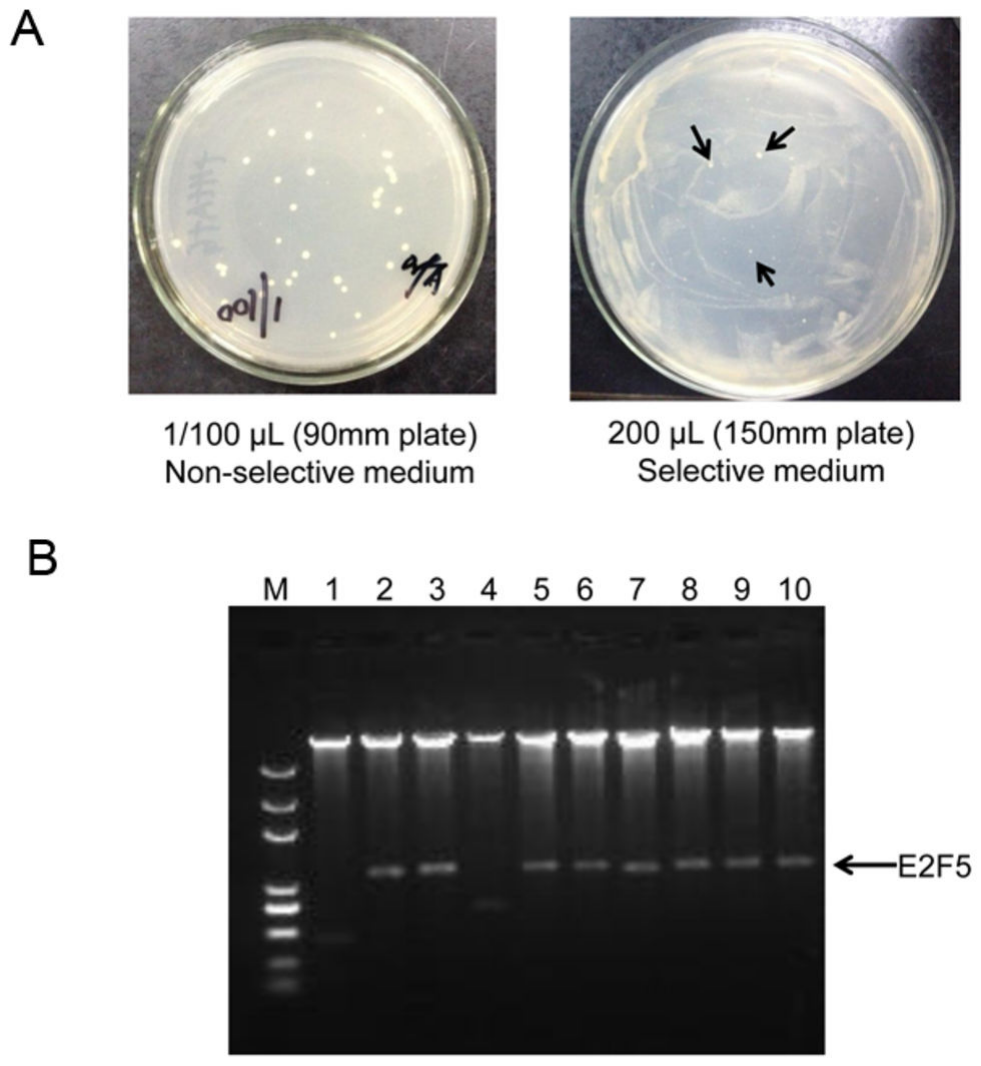
Specificity test for library screening via this system. (A) Performance of orfA screening in a library. Hybrid products were plated on non-selective and selective plates respectively. (B) Double digestion of library plasmids recovered from candidate yeast colonies. To determine the insert size in prey vectors, extracted plasmids from positive colonies were digested by *Bam*HI and *Xho*I.

 The colonies on non-selective plates were observed after mating 3 days. However, colonies in selective plates were observed after mating 7 days (Figure 5A). Ten colonies were isolated from selective plates, and library plasmids were identified from the positive colonies by amplification of Leu2 marker with primers LeuF/LeuR. The results of *Bam*HI/X*ho*I double digestion isolated prey vectors demonstrated that 8 of 10 colonies were similar with the size of E2F5 (Figure 5B). Also, the sequencing results confirmed that the 8 prey vectors were all pYE-Fok-E2F5. All these data indicated that E2F5 was screened from the library through this system, and the positive rate was about 80%. 

## Discussion

The identification of protein–protein interactions is critical to understand the complex functions of proteins and their networks in living cells. Many methods have been used to identify and characterize interactions between two or more proteins in diverse model organisms at the proteome-scale including *Saccharomyces cerevisiae* [22], *Caenorhabditis elegans* [23], *Drosophila melanogaster* [24], and humans [25]. There was a significant lack of concordance among the interaction data sets presented in these studies because of a limited number of protein interactions that were assayed, false positives, and false negatives [8]. In addition, with traditional Y2H screening methods, some detected interactions are unlikely to be biologically relevant because of the non-specific interactions that are introduced by Gal4 fusion bait and prey proteins.

In this study, we created an innovative approach to integrate modified ZFNs into the Y2H system to better identify protein–protein interactions. This approach is based on two characteristics of ZFNs. First, ZFNs are constituted of two separable protein domains—a DNA-binding domain (ZFP) and a non-specific DNA cleavage domain (FokI). Traditionally, ZFP and FokI are linked together in the constructs of ZFNs. We separated ZFP and FokI by fusing two proteins of interest. Upon the interaction of the two proteins, ZFNs will be constituted at its target site, which is provided on a reporter vector. The second feature of ZFNs is its sequence specificity when it functions as a DNA endonuclease. We put a ZFN target site, the BCR sequence, and a 6-bp spacer flanked by two 30-bp *Gal4* homology regions in the reporter vector. With ZFN function, a DSB is generated and will be repaired by SSA to restore Gal4 function, which subsequently drives reporter gene expression.

A fundamental concept of this system is that the reconstitution of functional ZFNs was solely dependent on specific protein interactions between proteins fused to ZFP and FokI. To this end, we took the advantage of two pairs of known interacting proteins: murine p53/SV40LT and human E2F5/WDSV orfA. The strong interaction between p53 and SV40LT was discovered two decades ago, and the interaction between human E2F5 and WDSV orfA was discovered in our lab. In our Y2H assay, the interactions of these two pairs of proteins successfully reconstituted the ZFN function. Therefore, a functional Gal4 drove the expression of the reporter genes of *His3*, *Ade2*, and *LacZ* in yeast cells as demonstrated in Figure 3A and 3B. Notably, less than 25.0% of transformants survived on selective medium (Figures 3C and 3D). The most reasonable explanation is that the long distance between the ZFP and FokI domains reduced the DNA cleavage efficiency of functional ZFNs. Moreover, no cross reactivity was observed between non-cognate protein pairs (Figures 3E and 3F).

A previous report suggested that the linkers between ZFP and FokI were less than 20 amino acids, and the DNA-binding efficiency of ZFP or ZFNs decreased with increasing linker length [26]. In this study, the pBCRZFN-53 plasmid contains a 957-bp truncated p53 sequence between the ZFP and FokI domains, and pBCRZFN-LT has a 1866-bp truncated SV40LT sequence. We extended the linkers to be as long as 622 amino acid residues and the modified ZFNs maintained their functions *in vivo*. We tested the activities of different ZFNs in which proteins of interest were fused with both sites (N terminal or C terminal) of ZFP and FokI, However, the result demonstrated that either N or C terminal site didn’t not influence functions of ZFNs (data not shown).

In previous experiments, we found that the frequency of *Gal4* spontaneous restoration events was approximately 1 × 10^-6^ in yeast strain AH109. Notably, because of the limited transformation efficiency, no transformants of the reporter vector pADH-Gal4-BS only or co-transformants of pADH-Gal4-BS and empty expression vectors survived on the selective medium. Even though the number of transformants was up to 1 × 10^6^, only one false positive was obtained in this system. Moreover, interaction between WDSV orfA and E2F5 could be identified by screening cDNA library against orfA protein, and the positive rate was as high as 80%. However, all screened E2F5 were in the format of plasmids pYE-Fok-E2, not in the cDNA library. This is due to the fact that plasmid pYE-Fok-E2 is more abundant than E2F5 in the cDNA library. Thus, the extraordinarily low false-positive rate has very little negative impact on the screen for protein interactions.

We were interested in generating an alternative system to explore novel protein interactions with a low false-positive rate. To this aim, we designed a Y2H system that used the Gal4 transcription factor as the reporter molecule, avoiding Gal4-fusion proteins in bait or prey that activate the expression of reporter genes in the absence of specific protein interactions. The traditional Y2H method always leads to a high false-positive rate, which has impeded the development of a suitable Y2H system. In contrast, our novel Y2H system has the ability to reduce the false-positive rate to values as low as 10^-6^. Moreover, this ZFN-Y2H method is robust enough to discover new protein interactions and help researchers to better understand protein interactomics.

## Supporting Information

Figure S1 **Schematic diagram of ZFN expression plasmid with different spacers.** Plasmid pBCR-ZFN expresses conventional ZFN contains a short spacer( 4~6 amino acids ) between ZFP and FokI domain. For insertion of long spacers between ZFP and FokI, either *p53* or *SV40LT* DNA fragments were cloned into pBCR-ZFN between *Bam*HI and *Spe*I sites, resulting in plasmids pBCRZFN-53 or pBCRZFN-LT. NLS, SV40 nuclear localization signal; p53, truncated murine p53 (a.a. 72–390); SV40LT, SV40 large T antigen (a.a. 87-708); T_CYC3_: yeast cyc3 terminator.
(TIF)

Figure S2 **Schematic diagram of ZFP-fusion expression plasmid.** Parent plasmid pRS-ADH contains a multiple clone site (MCS) for foreign genes clone. Firstly, DNA fragments of BCR-ZFP were inserted into pRS-ADH to generate pRS-ZFP. Then, SV40LT and WDSV-orfA were respectively amplified and cloned into pRS-ZFP between *Eco*RI and *Xho*I, resulting in plasmids pRS-ZFP-LT and pRS-ZFP-A.
(TIF)

Figure S3 **Schematic diagram of FokI-fusion expression plasmid.** PCR products of FokI were cloned into parent plasmid pYE-ADH between *Xba*I and *Bam*HI sites. P53 and was cloned into N-terminus of FokI between *Eco*RI and *Sal*I, and human E2F5 was inserted into pYE-FokI between *Sac*II and *Nco*I site, respectively. FokI-p53 and FokI-E2F5 fusion proteins expression was under the control of ADH1 promoter.
(TIF)

Figure S4 **Schematic of reporter plasmid and Gal4 expression vector.** Basing on transcriptional factor Gal4, we developed a reporter vector pADH-Gal4-BS containing three key elements, *KanMX4* expression cassette, *CEN/ARS* replication origin and Gal4BD-AD expression cassette. *KanMX4* as a marker gene provides yeasts have the ability to survive in the presence of G418 in medium. *CEN/ARS* replication origin maintained reporter plasmids harboring ZFN target sequence at one or two copies in yeast nucleus. In reporter, a ZFN-binding site (BS) flanking two 30bp-repeats of *Gal4BD* sequence, and a stop codon TAA in the spacer of BS. Thus, the reporter plasmid expresses dysfunctional Gal4 without ZFN cleavage. However, in the presence of ZFN target at BS, a repeat and BS are removed in the process of DNA repair. Subsequently, the repaired reporter plasmid expresses functional Gal4 protein to drive *His3*, *LacZ* expression in AH109. In addition, control vector pADH-Gal4 contains wildtype *Gal4BD-AD* sequence, and expresses functional Gal4 directly. Both of these two plasmids were constructed in our previous study. Plasmid pADH-Gal4-MCS is a parent plasmid for p ADH-Gal4-BS, in which ZFN binding site was cloned between *Not*I and *Bam*HI sites.
(TIF)
