# Supplementary figures and images for: A Novel Genetic System Based on Zinc Finger Nucleases for the Identification of Interactions between Proteins In Vivo
Source: PLoS One. 2013 Dec 31;8(12):e85650. doi: 10.1371/journal.pone.0085650 (PMC3877365; doi:10.1371/journal.pone.0085650)

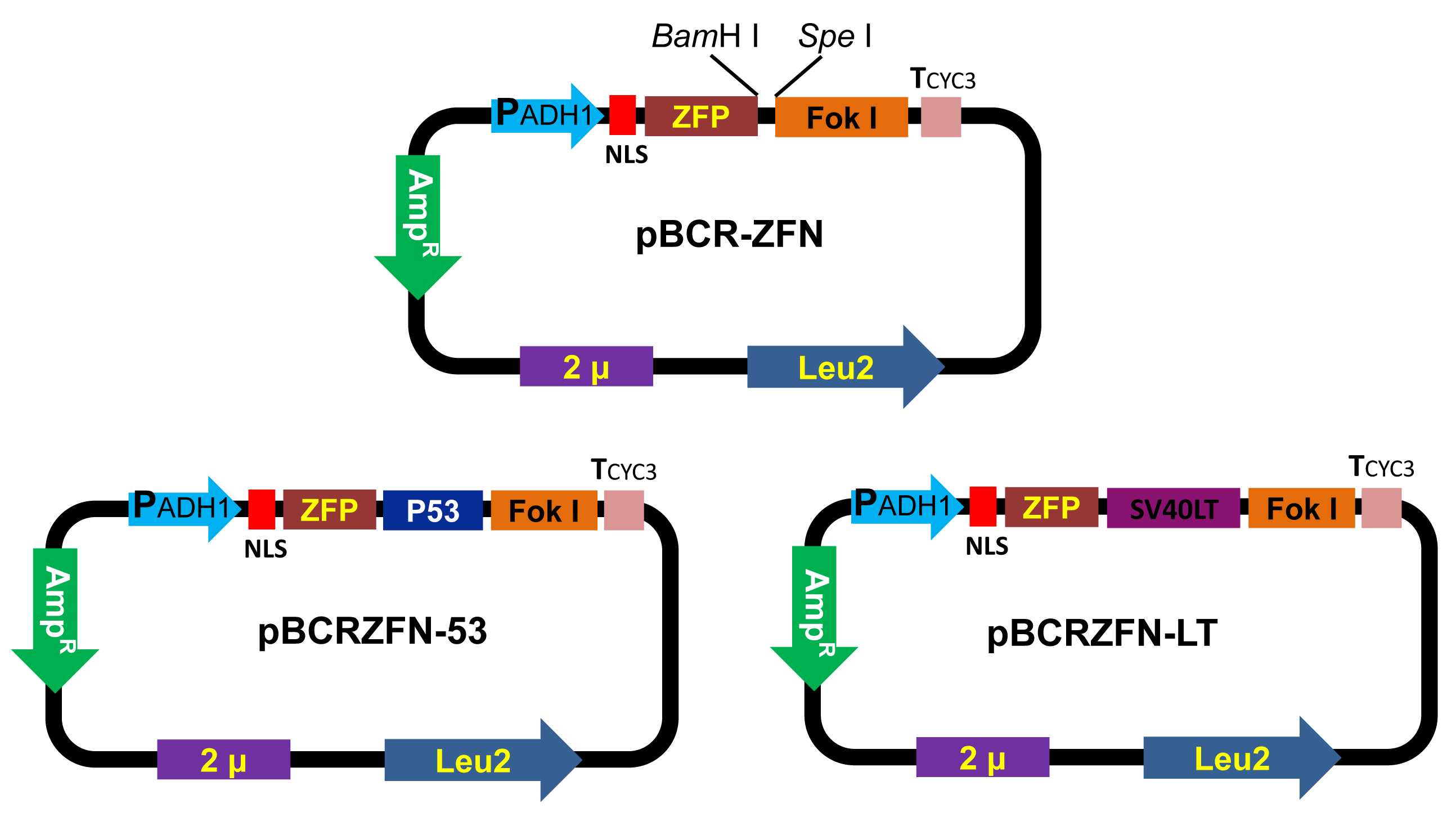

Supplement: Figure S1 — Schematic diagram of ZFN expression plasmid with different spacers. Plasmid pBCR-ZFN expresses conventional ZFN contains a short spacer( 4~6 amino acids ) between ZFP and FokI domain. For insertion of long spacers between ZFP and FokI, either p53 or SV40LT DNA fragments were cloned into pBCR-ZFN between BamHI and SpeI sites, resulting in plasmids pBCRZFN-53 or pBCRZFN-LT. NLS, SV40 nuclear localization signal; p53, truncated murine p53 (a.a. 72–390); SV40LT, SV40 large T antigen (a.a. 87-708); TCYC3: yeast cyc3 terminator. (TIF) [file pone.0085650.s001.tif]

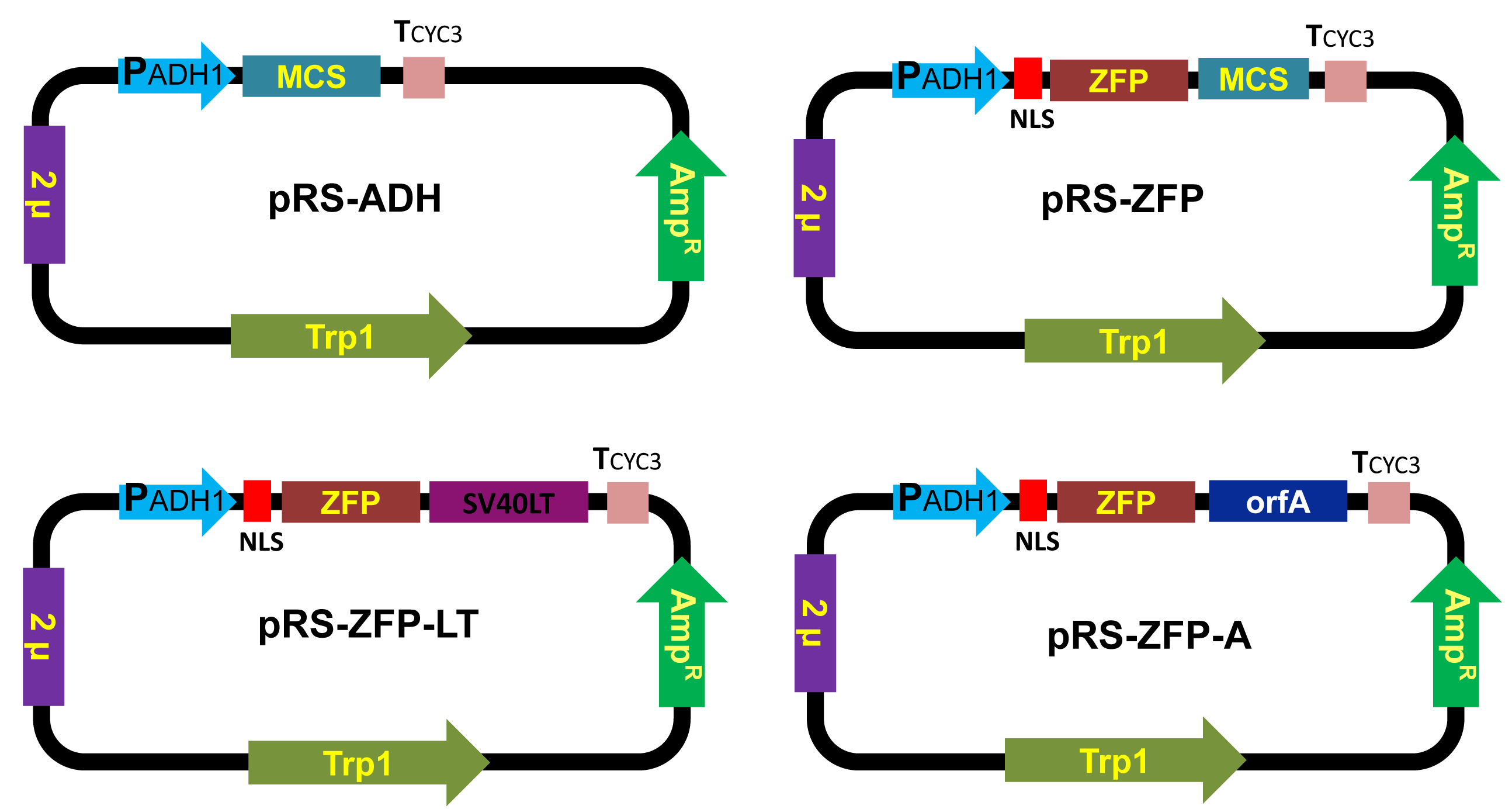

Supplement: Figure S2 — Schematic diagram of ZFP-fusion expression plasmid. Parent plasmid pRS-ADH contains a multiple clone site (MCS) for foreign genes clone. Firstly, DNA fragments of BCR-ZFP were inserted into pRS-ADH to generate pRS-ZFP. Then, SV40LT and WDSV-orfA were respectively amplified and cloned into pRS-ZFP between EcoRI and XhoI, resulting in plasmids pRS-ZFP-LT and pRS-ZFP-A. (TIF) [file pone.0085650.s002.tif]

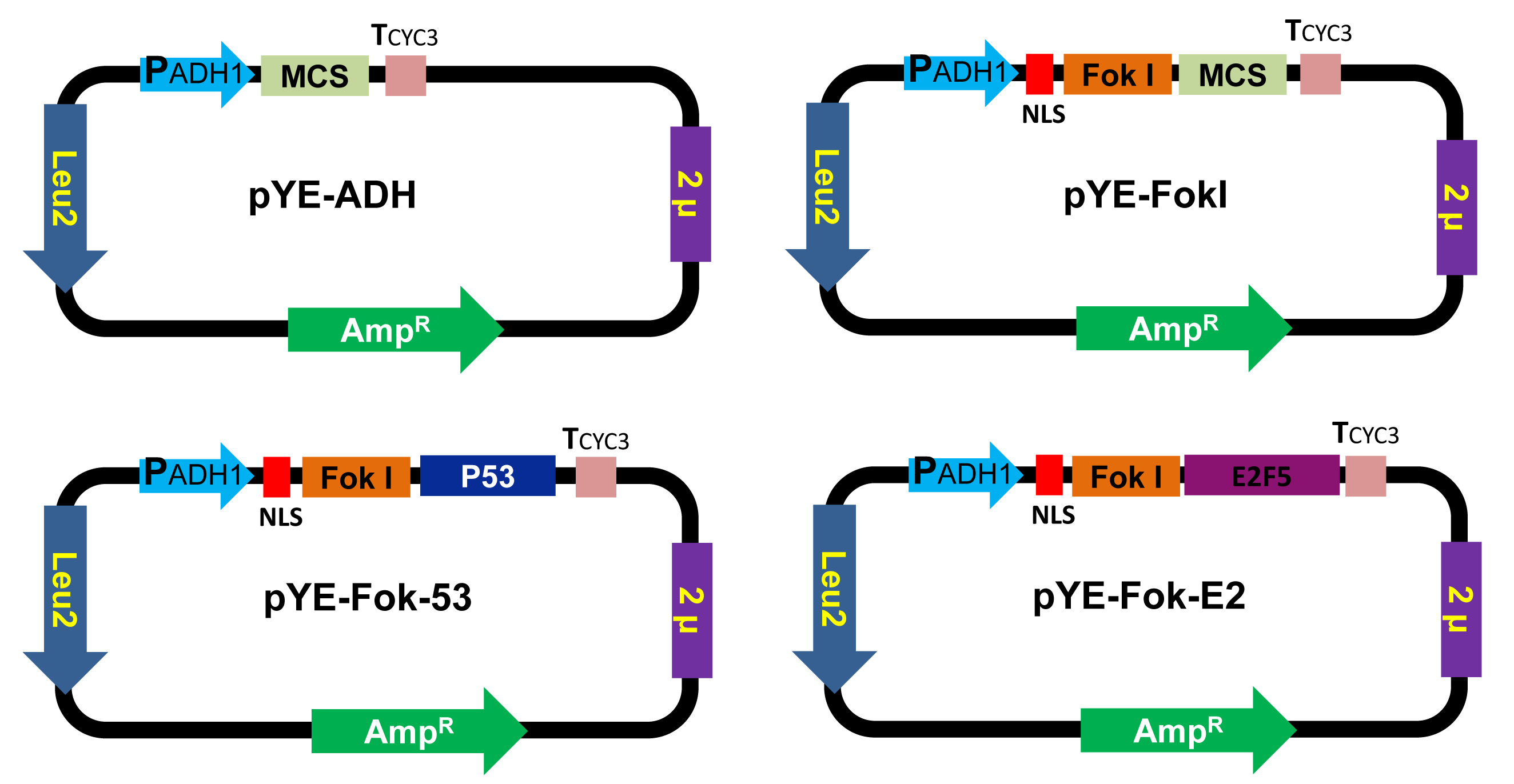

Supplement: Figure S3 — Schematic diagram of FokI-fusion expression plasmid. PCR products of FokI were cloned into parent plasmid pYE-ADH between XbaI and BamHI sites. P53 and was cloned into N-terminus of FokI between EcoRI and SalI, and human E2F5 was inserted into pYE-FokI between SacII and NcoI site, respectively. FokI-p53 and FokI-E2F5 fusion proteins expression was under the control of ADH1 promoter. (TIF) [file pone.0085650.s003.tif]

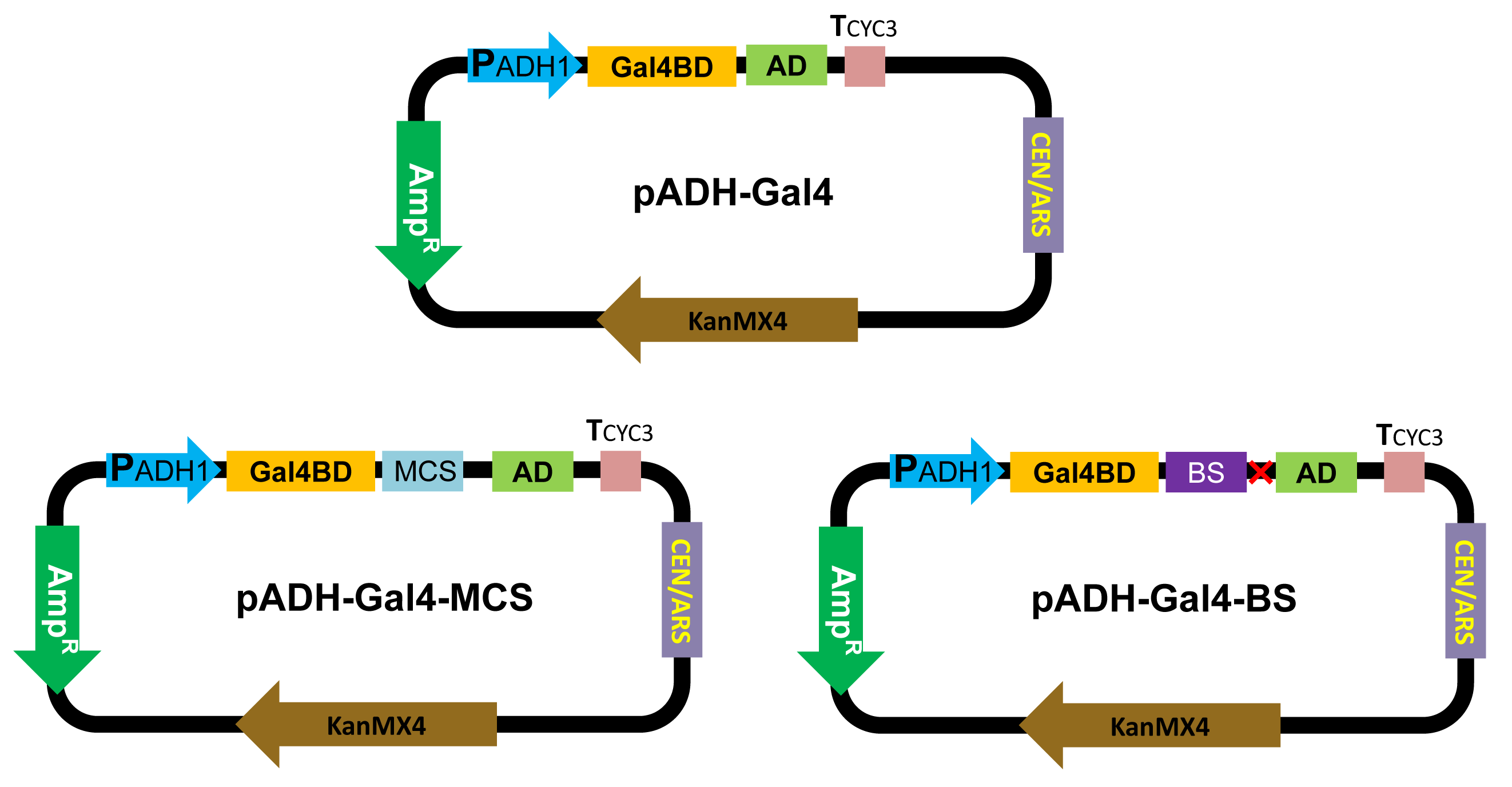

Supplement: Figure S4 — Schematic of reporter plasmid and Gal4 expression vector. Basing on transcriptional factor Gal4, we developed a reporter vector pADH-Gal4-BS containing three key elements, KanMX4 expression cassette, CEN/ARS replication origin and Gal4BD-AD expression cassette. KanMX4 as a marker gene provides yeasts have the ability to survive in the presence of G418 in medium. CEN/ARS replication origin maintained reporter plasmids harboring ZFN target sequence at one or two copies in yeast nucleus. In reporter, a ZFN-binding site (BS) flanking two 30bp-repeats of Gal4BD sequence, and a stop codon TAA in the spacer of BS. Thus, the reporter plasmid expresses dysfunctional Gal4 without ZFN cleavage. However, in the presence of ZFN target at BS, a repeat and BS are removed in the process of DNA repair. Subsequently, the repaired reporter plasmid expresses functional Gal4 protein to drive His3, LacZ expression in AH109. In addition, control vector pADH-Gal4 contains wildtype Gal4BD-AD sequence, and expresses functional Gal4 directly. Both of these two plasmids were constructed in our previous study. Plasmid pADH-Gal4-MCS is a parent plasmid for p ADH-Gal4-BS, in which ZFN binding site was cloned between NotI and BamHI sites. (TIF) [file pone.0085650.s004.tif]
